# Accelerated loss of trunk muscle density and size at L1 vertebral level in male patients with COPD

**DOI:** 10.3389/fendo.2022.1087110

**Published:** 2022-12-15

**Authors:** Ying Wang, Sidong Li, Zhenyi Zhang, Shiqi Sun, Juntao Feng, Jinbiao Chen, Yigang Pei, Xianjing Peng

**Affiliations:** ^1^ Department of Radiology, Xiangya Hospital, Central South University, Changsha, China; ^2^ National Clinical Research Center for Geriatric Disorders, Xiangya Hospital, Changsha, China; ^3^ Division of Life Sciences and Medicine, University of Science and Technology of China, Hefei, China; ^4^ Department of Radiology, Taojiang County People’s Hospital, Yiyang, China; ^5^ Department of Respiratory Medicine, Xiangya Hospital, Central South University, Changsha, China; ^6^ Department of Medical Records & Information, Central South University, Changsha, China

**Keywords:** chronic obstructive pulmonary disease, L1-trunk muscle, muscle size, muscle density, change

## Abstract

**Background and purpose:**

Weight loss and muscle mass loss are common in patients with chronic obstructive pulmonary disease (COPD). Muscle density and fat infiltration based on CT images may be more sensitive than muscle mass by DXA in the assessment of sarcopenia for COPD patients. However, the age-related changes of cross-sectional trunk muscle compositions based on lung CT scans are still unknown. Thus, we aimed to investigate over time the change in muscle density, size, and fat deposition of L1-level trunk muscles in patients with COPD.

**Materials and methods:**

129 male COPD patients with a second chest CT scan (from 2013-2019 to 2014-2020) were enrolled. The CT images at first and second CT scans are analyzed by OsiriX software. Trunk muscles at the level of the 1st lumbar vertebrae were selected for analysis. Attenuation of lumbar vertebrae 1 was also measured from chest CT images. The pulmonary function values were calculated based on forced expiratory volume in 1 second (FEV1) and forced vital capacity (FVC).

**Results:**

The mean age of the 129 patients with COPD was 69.7 years. The durations of COPD of this cohort were from 8-17 years. The mean area and density of L1 trunk muscles were 85.5 cm^2^ and 36.4 HU. At baseline, muscle area and density and vertebral density were negatively associated with age (p<0.0001), while the intermuscular fat area and the fat infiltration ratio were not significantly associated with age (p>0.05). The per-year loss of trunk muscle area was 2.83 cm^2^ (p<0.0001) which accounts for 3.3% decrease per year, and the per-year decrease of trunk muscle density was 2.41 HU (p<0.0001) which accounts for 6.6% decrease per year. The per-year increase of intermuscular fat in trunk muscles was 0.57 cm^2^ (p=0.006) which accounts for 11.1% increase per year. The bone density loss was 5.63 HU/per year (p<0.0001).

**Conclusion:**

Men with COPD had accelerated muscle loss as well as increased fat infiltration. Compared to muscle quantity loss, the decline in muscle quality is much larger, indicating the importance of relevant interventions focusing on improving muscle quality.

## Introduction

Chronic obstructive pulmonary disease (COPD) is the fourth leading cause of death worldwide and is often characterized by chronic inflammation and extrapulmonary changes that impair quality of life and physical activity (1). Thus, weight loss and muscle mass loss are common in patients with COPD (2), and the risk of developing sarcopenia in COPD patients is increasing, with a prevalence ranging from 15% to 55% (3). Furthermore, sarcopenia appears to negatively affect clinical outcomes related to function and health in patients with COPD (4). Therefore, early detection of sarcopenia might be critical to better designing therapeutic interventions like pulmonary rehabilitation.

Skeletal muscle mass is usually measured by dual energy X-ray absorptiometry (DXA) and bioelectrical impedance analysis (BIA). Unlike DXA/BIA, which only measures muscle quantity, CT measures both muscle quantity (e.g., muscle cross-sectional area [CSA] or muscle index) and muscle quality (e.g, muscle attenuation or fat infiltration) (5). CT-based muscle metrics show promise in predicting the risk of osteoporotic fracture (6–8) and hip refracture (9), as well as the mortality (10–12). Muscle density and fat infiltration may be more sensitive than muscle mass by DXA in the assessment of sarcopenia for COPD patients.

In clinical practice, routine lung CT is often performed in COPD patients to help characterize COPD phenotypes and screen for lung cancer (13). Therefore, compared with DXA, the use of lung CT might offer the opportunity to routinely assess the sarcopenia in COPD patients. However, the age-related changes of cross-sectional trunk muscle compositions based on lung CT scans are still unknown, which impedes the understanding of the relationships between muscle metrics and COPD.

In this retrospective follow-up cohort study, by using state-of-the-art imaging, we aimed to investigate over time the change in muscle density, size, and fat deposition of L1-level trunk muscles in patients with COPD. We also aimed to explore the relations among muscle measurements, duration of COPD, and pulmonary function.

## Materials and methods

### Participants

The retrospective study was approved by the Institutional Review Board of Xiangya Hospital, and the informed consent from the patients was waived. We searched both the medical record and the PACS for all patients who met the following inclusion criteria: a male aged 50 years or older with a diagnosis of COPD according to the Global Initiative for Chronic Obstructive Lung Disease (GOLD) criteria (14) between Jan 1, 2013, and Dec 31, 2019; two diagnostic chest CT scans with an interval time of at least 3 months. This yielded 129 male patients with a second chest CT scan (from 2013-2019 to 2014-2020).

Patients with other conditions that cause sarcopenia including chronic liver disease, end stage renal disease, and active cancer were excluded. Bronchial asthma, interstitial pneumonia, or bronchiectasis, thoracic or upper lumbar vertebrae degenerative disease or history of operation on the vertebrae, without a second chest CT scan were also excluded.

### CT scans

CT imaging was performed with two CT scanners (Aquilion one 320, Toshiba, Tokyo, Japan; SOMATOM Definition, Siemens, Erlangen, Germany). All patients were scanned in the supine position during deep inspiration without intravenous contrast. The two scans of most COPD patients were performed on a same scanner to avoid the inter-scanner differences. Scan parameters were 120kVp, auto current setting based on BMI, 1mm slice thickness, 50 cm field of view, and 512×512 matrix in spiral and standard reconstructions.

### Muscle and bone measurements

**Figure 1:**
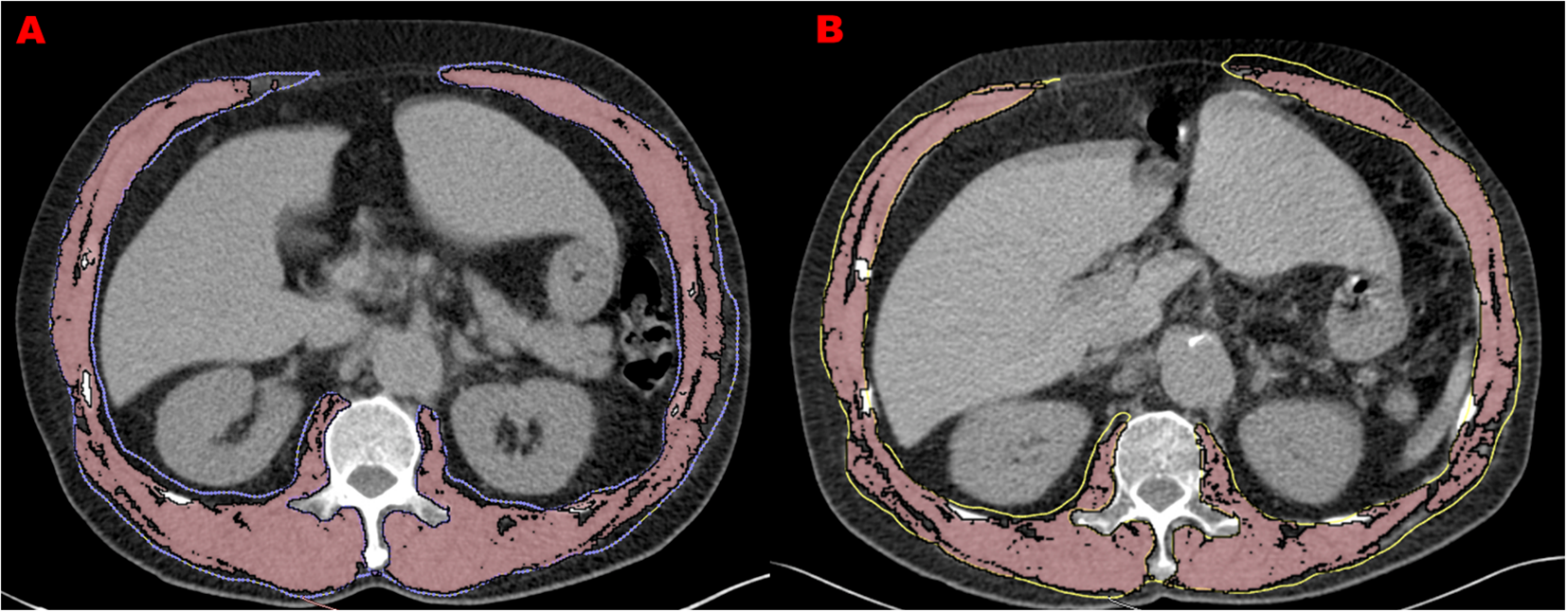
Muscle measurements at two screening chest CTs in 66-year-old man with COPD. **(A)** refers to L1 level CT image of 1^st^ CT scan with the measurement of trunk muscle density (31.9 HU) and size (140.7 cm^2^). **(B)** refers to L1 level CT image of 2^nd^ CT scan performed 3 years after the initial one with the measurement of trunk muscle density (26.1 HU) and size (114.8 cm^2^).

The DICOM images of COPD patients at first and second CT scans are analyzed by OsiriX software (Lite version 10.0.2, Pixmeo, Geneva, Switzerland) which was downloaded from http://www.osirix-viewer.com/, and was previously assessed as a user-friendly image analysis software package for the Apple Mac OS. All muscle measurements were acquired by one of the investigators who, in preparation for the measurements, received training in chest CT imaging assessments focusing on trunk muscle morphology. For practice purposes, a sample of about 20 images was analyzed with the OsiriX software application prior to the beginning of the measurement study. Trunk muscles at the level of the 1st lumbar vertebrae were selected for analysis. Using the ‘pencil’ tool to outline the muscles as ROIs. Then open the ‘growing’ window, -29 to 150 HU as the threshold to segment muscle tissue from fat (**Figures 1A, B**). The area and mean CT values were displayed in the Segmentation preview box. The pre-segmentation ROI was defined as “Muscle Fat”, and the area of pre-segmentation ROI minus the area of segmented muscle was the fat area (intermuscular fat).

Attenuation of lumbar vertebrae 1 (L1) was also measured by the same investigator with the OsiriX software application from chest CT images. A circular region of interest (ROI) was drawn at the mid-vertebral body, avoiding the cortical bone and the posterior internal vertebral venous plexus.

### Pulmonary function testing and other covariates

The pulmonary function values were calculated based on forced expiratory volume in 1 second (FEV1) and forced vital capacity (FVC) by using standardized equipment (MasterScreen, JAEGER/Carefusion, Hoechberg, Germany).

Demographic and anthropometric covariates included age, height, weight, duration of COPD. Health-related covariates included hemoglobin, mean hemoglobin concentration, mean corpuscular hemoglobin, white blood cell count, red blood cell count, platelet distribution width, mean platelet volume.

### Statistical analysis

Baseline characteristics were described with mean ± standard deviation for continuous variables and frequency and percent for categorical variables. Generalized linear models were used to study the association of muscle indexes with age, duration of disease, and pulmonary function. Moreover, we assessed the impact of COPD duration, age, and pulmonary function on muscle indexes by categorizing participants with COPD using 10 years, age using 70 years, and FEV1% using 40 as the cut-off value. Comparisons were made by two-sample t-test or Wilcoxon Signed Rank tests for continuous variables.

A two-sided P value less than 0.05 was considered statistically significant. All analyses were performed using SAS 9.4.

## Results

### Baseline parameters

The demographic data and baseline measurements were shown in **Table 1**. The mean age of the 129 patients with COPD was 69.7 years. The durations of COPD of this cohort were from 8-17 years. The mean area and density of L1 trunk muscles were 85.5 cm^2^ and 36.4 HU, and the mean bone attenuation of L1 was 113.8 HU. This COPD cohort had a mean FEV1 of 40.3%. The blood routine examinations of this cohort were normal.

**Table 1:** Characteristics of study participants at baseline (N=129).

	Mean ± SD
Age, yrs	69.7 ± 10.0
Height, cm	164.2 ± 6.8
Weight, kg	56.1 ± 9.8
Duration of disease, yrs	10 (8-17)
Muscle and bone indexes	
Muscle Fat Area, cm^2^	93.9 ± 20.8
Muscle area, cm^2^	85.5 ± 18.0
Fat area, cm^2^	8.8 ± 5.4
Fat infiltration, %	0.09 ± 0.04
Muscle density, HU	36.4 ± 6.9
Vertebrae density, HU	113.8 ± 43.9
Blood routine examination	
Hemoglobin, g/L	128.9 ± 21.2
Mean hemoglobin concentration, g/L	325.7 ± 11.3
Mean corpuscular hemoglobin, pg	30.4 ± 2.1
White blood cell count, 10^9/L	7.3 ± 2.7
Red blood cell count, 10^12/L	4.3 ± 0.6
Platelet distribution width, %	13.8 ± 3.2
Mean platelet volume, fL	9.6 ± 1.2
Pulmonary function*	
FEV1, L	0.98 (0.61-1.30)
FEV1, %	40.3 (24.5-60.2)
FVC, L	2.4 (2.0-2.8)
FVC, %	77.0 (65.9-96.9)
FEV1/FVC	0.37 (0.28-0.51)

*Pulmonary function was available for 84 patients.

FEV1, forced expiratory volume in the first second; FVC, forced vital capacity.

### Associations of muscle and bone indexes with age, duration of COPD and pulmonary function

**Figure 2:**
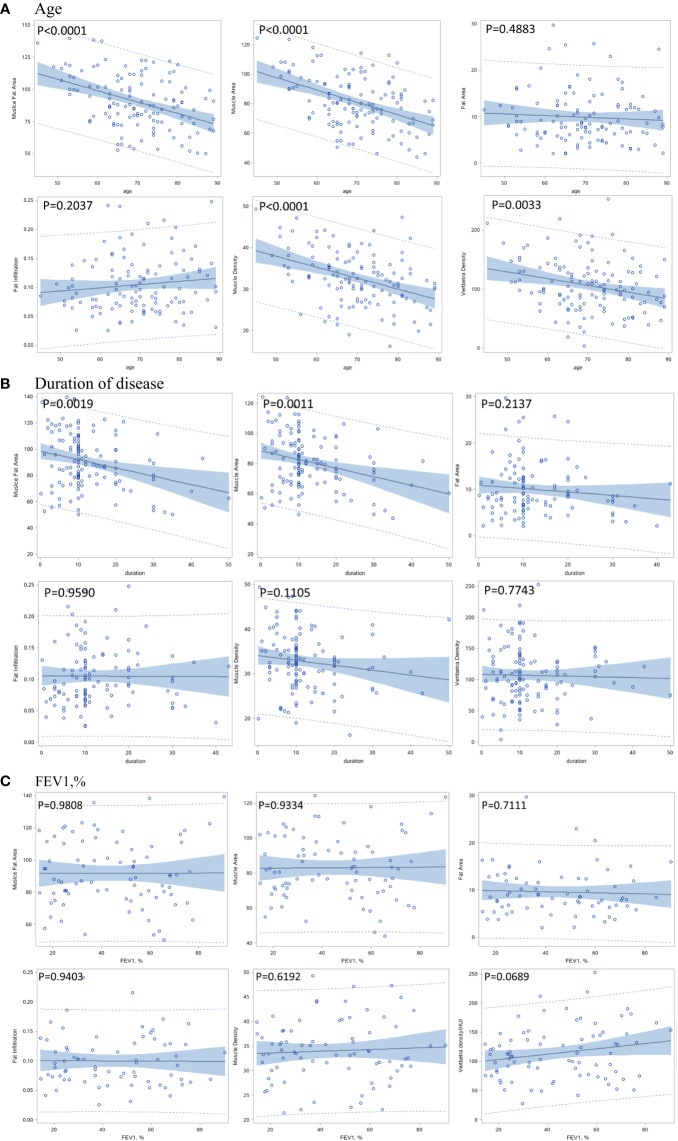
Correlation between baseline muscle and bone indexes with age **(A)**, duration of disease **(B)**, and pulmonary function **(C)**.

At baseline, **Figure 2A** shows that muscle area and density and vertebral density were negatively associated with age (p<0.0001), while the intermuscular fat area and the fat infiltration ratio were not significantly associated with age (p>0.05). The duration of COPD was only negatively associated with muscle area (p=0.0011), but was not associated with muscle and vertebral density (**Figure 2B**). **Figure 2C** shows that FEV1, % was not significantly associated with any measurement.

**Figure 3:**
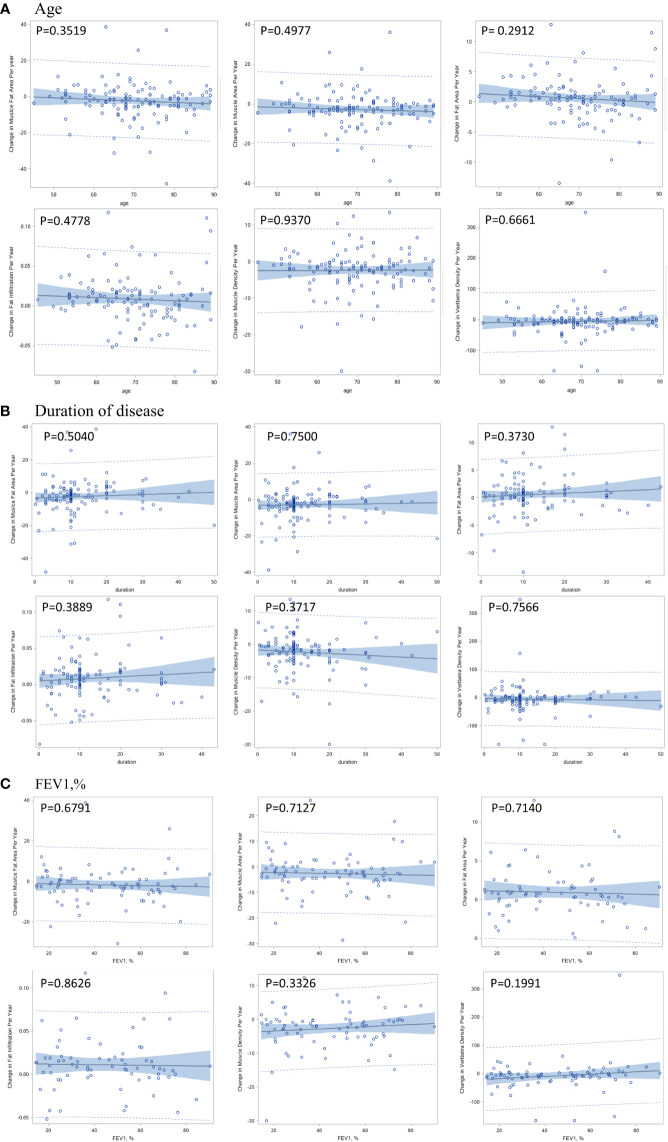
Correlation between per-year change in muscle and bone indexes with age **(A)**, duration of disease **(B)**, and pulmonary function **(C)**.

The per-year changes in muscle and bone indexes were not significantly associated with age, duration of COPD and FEV1, % (**Figures 3A–C**).

### Per-year changes of muscle metrics

**Table 2** shows the absolute changes and per year changes in muscle and bone indexes between the two CT scans for the male COPD patients. The per-year loss of trunk muscle area was 2.83 cm^2^ (p<0.0001) which accounts for 3.3% decrease per year, and the per-year decrease of trunk muscle density was 2.41 HU (p<0.0001) which accounts for 6.6% decrease per year. The per-year increase of intermuscular fat area in trunk muscles was 0.57 cm^2^ (p=0.006) which accounts for 11.1% increase per year. For male COPD patients, the bone density loss was 5.63 HU/per year (p<0.0001). For the subgroup analysis, the group aged under 70 years had a higher intermuscular fat area (0.98 cm^2^) increase per year compared to that of the group aged over 70 years (0.09 cm^2^). However, the difference of muscle fat infiltration per year between two age groups was border significant (p=0.04). The COPD duration and the pulmonary function had no effect on the per-year change of all muscle indexes (**Table 3**).

**Table 2:** Per-year Change in muscle and bone indexes in COPD patients.

	First measurement	Second measurement	Change	Per year change (n%)	P value
Muslce Fat Area, cm^2^	93.95 ± 20.94	90.28 ± 20.91	-3.67 ± 8.75	-2.36 ± 10.29(-2.5%)	<0.0001
Muscle area, cm^2^	85.54 ± 18.07	81.1 ± 17.86	-4.44 ± 7.67	-2.83 ± 8.73(-3.3%)	<0.0001
Fat area, cm^2^	9.04 ± 5.42	9.9 ± 5.59	0.85 ± 3.26	0.57 ± 3.37(6.3%)	0.006
Fat infiltration, %	0.09 ± 0.04	0.1 ± 0.05	0.01 ± 0.03	0.01 ± 0.03(11.1%)	<0.0001
Muscle density, HU	36.53 ± 6.97	32.73 ± 6.55	-3.8 ± 5.59	-2.41 ± 5.64(6.6%)	<0.0001
Vertbetra density, HU	114.14 ± 43.93	106.6 ± 44.06	-7.55 ± 29.96	-5.63 ± 47.8(4.9%)	<0.0001

Analysis was conducted among patients with complete information on muscle and bone indexes in two measurements. Comparisons between two measurements were conducted using paired t tests and signed rank tests according to the normality of distribution.

**Table 3:** Per-year change in muscle indexes in subgroups.

	Disease duration			Age			FEV1%		
	<=10 yrs (n=75)	>10 yrs (n=50)	P value	<=70 yrs (n=67)	>70 yrs (n=59)	P value	<40 (n=39)	>=40 (n=42)	P value
Muslce Fat Area, cm^2^	-3.19 ± 10.71	-1.13 ± 9.71	0.14	-1.61 ± 9.32	-3.21 ± 11.31	0.29	-0.96 ± 9.51	-2.61 ± 8.57	0.33
Muscle area, cm^2^	-3.25 ± 9.18	-2.22 ± 8.15	0.21	-2.41 ± 7.67	-3.3 ± 9.84	0.86	-1.82 ± 7.81	-3.32 ± 7.69	0.36
Fat area, cm^2^	0.32 ± 3.24	0.98 ± 3.59	0.99	0.98 ± 3.27	0.09 ± 3.45	0.01	1.19 ± 3.19	0.73 ± 2.87	0.65
Fat infiltration, %	0.01 ± 0.03	0.01 ± 0.04	1.00	0.01 ± 0.03	0.01 ± 0.03	0.04	0.01 ± 0.03	0.01 ± 0.03	0.70
Muscle density, HU	-1.73 ± 5.21	-3.36 ± 6.18	0.21	-2.59 ± 6.16	-2.2 ± 5.03	0.86	-3.05 ± 6.58	-2.34 ± 5.05	0.84

Analysis was conducted among patients with complete information on muscle indexes in two measurements. Comparisons between two groups were conducted using Wilcoxon two-Sample tests.

## Discussion

To the best of our knowledge, our findings first demonstrated the rate of decline in muscle size and density in older COPD patients, and we discovered that older male COPD patients had accelerated loss of muscle size and density, as well as increased intermuscular fat deposits. Our results also indicate that compared to muscle quantity decreased, the rate of muscle quality loss is doubled, implying that the interventions for sarcopenia of COPD patients should be focused on muscle quality.

In the last decade, there has been a rapid increase in the use of CT measured muscle CSA and density in the evaluation of sarcopenia, especially for opportunistic applications. A recent review showed that the total abdominal wall muscles are mostly favored for muscle CSA and density measurements, and measuring trunk muscle in an axial slice is the best standard for CT-based calculation of total body muscle mass (15). Further, a recent opportunistic use of CT study demonstrated that the L1 trunk muscle measurement allows sarcopenia assessment using both chest and abdominal CT scans (11), which indicates our outcomes could generate the potential yield of opportunistic CT screening of sarcopenia for COPD patients.

The decline rate of muscle mass in older adults was reported as approximately 0.51%, which is much lower than that of muscle strength, which was 2.5 to 4% in a year (16). The cross-sectional results showed that muscle mass decreased less than 0.5% per year in both genders when compared the young adults (18 to 45 years old) and the elderly (65 years old or over) (16). However, our results indicated that the muscle size loss in older male patients with COPD was 3.3% in a year, which is much higher than older adults without COPD.

Inflammation is the main feature of chronic obstructive pulmonary disease. Usually, the inflammatory response of COPD patients is not limited to the lungs but is also accompanied by systemic chronic inflammation (1). Systemic chronic inflammatory responses can lead to sarcopenia in COPD patients (1). On the other hand, sarcopenia was found to be associated with increased levels of systemic inflammation in COPD patients (17). In addition to the inflammation effect, physical inactivity caused by COPD is also a critical trigger for muscle loss as inactivity results in disuse-atrophy (3). Thus, in our study, the muscle loss of COPD patients was much larger than the numbers in the reports with normal elderly men without COPD.

The observations of larger declines of muscles, especially the muscle density, are meaningful because previous reports have demonstrated reduced attenuation in the range of 3–6 HU among people with strength deconditioning and low back pain (18, 19). Furthermore, the 2.41 HU decrease in COPD men per year is parallel to a 15% decrease over four decades in a previous report (20). Notably, the mean value (36.9HU) of muscle density in our study was adjacent to the myosteatosis diagnostic cut points (from ≤22.0 HU to ≤44.4 HU) for muscle attenuation in cancer patients (12). L1 trunk muscle density may be a potential risk factor for mortality in patients with COPD as other reports (14, 21).

Insight of the muscle size and density decreases and increased fat infiltration observed among male COPD patients in this study is also important to recognize the potential for intervention strategies that could be implemented to mitigate these detriments to muscle health. A study ever showed that muscle quality is more impaired in COPD patients with sarcopenia (22). Our results might indicate the importance of a rapid increase in muscle quality favoring a combination of resistance and aerobic exercises as commonly applied in pulmonary rehabilitation. Exercise training programs are of high importance, and it is a limitation of our study that we did not assess routine physical activity and exercises in this cohort.

### Limitations

Our study has several limitations. First, not all participants were stable COPD patients. Some of them came to our hospital for the second CT scan because of the aggravating COPD for in-hospital treatments. Second, we did not perform a detailed assessment of physical function, which may have added to a better understanding of the causes of accelerated loss of muscle in COPD patients. Third, we did not collect information about smoking history, which is known to affect muscle loss (23). Forth, the inflammation related information is not available in this study. One cause of the COPD related muscle loss is the inflammation status of the COPD patients.

In conclusion, COPD men had accelerated muscle loss and increased muscle fat infiltration. Compared to muscle size loss, the decline in muscle density was much larger, indicating the importance of relevant interventions focusing on improving muscle quality.

## Data availability statement

The raw data supporting the conclusions of this article will be made available by the authors, without undue reservation.

## Ethics statement

The studies involving human participants were reviewed and approved by Medical Ethics Committee of Xiangya Hospital of Central South University. Written informed consent for participation was not required for this study in accordance with the national legislation and the institutional requirements.

## Author contributions

All authors contributed to this study. YW: Designing the study and preparing the initial draft. ZZ: Data collect and analysis. SL: Data analysis and interpretation. JF and JC: Data acquisition, data analysis. YP: Critically revising the final manuscript. XP: Designing the study, conceptualization and critical revision for intellectual content. All authors contributed to the article and approved the submitted version.
